# The Mind Kills the Body—Extract

**Published:** 1874-09

**Authors:** J. C. Berry

**Affiliations:** West Virginia


### THE MIND KILLS THE BODY—EXTRACT.

BY J. C. BERRY, M.D.

*Read Before the State Medical Society of West Virginia.*

### *SPECIAL SENSES.*

I am in possession of five senses, and through them my existence is known to myself.

*Sense of Touch.*—I consider the sense of touch a common sensation, which is more highly developed in some particular parts—as the eyes, the auditory canal and its appendages, the olfactory region, or canal, and the delicate mucous membrane of the tongue and fauces; and these four are special only in their peculiar functions of transmitting light, sound, smell, etc.

I place an object upon my cheek, and the impression is conveyed to the brain through the sense of touch; I move it until it comes in contact with the retina, and the impression is conveyed through the same sense. Again, by bringing an article of food in contact with the tongue, I am able to distinguish its peculiar taste from that of another, this being its special function, but let me apply heat or cold, and I receive the impression through its common functions. I am able, through the sense of smell, to distinguish the fragrance eminating from flowers; I am able, through the sense of hearing, to distinguish sound, whether mild or harsh, pleasant or unpleasant; but obstruct or injure them in any way, and the impression is conveyed through their common functions. Hence, I could not have known life in its full and complete sense, had I

not first been provided with all these senses, which hold me in relation to external things—connecting the inner life to the outer.

How much we can do toward curing and relieving disease, through the special functions (of the five senses), without the use or application of medicines, I am not prepared to say; but it must be conceded that our success depends largely upon our *personal relations.* I do not adhere to the doctrine of Swedenborg, but I believe there is truth in what that extraordinary writer says in regard to "personal atmospheres." There are some physicians, and many men and women, whose presence is health-giving, exhilarating and comforting. The Rev. Dr. Eddy says: "So much importance do I attach to a life-full, rich, genial personality in a physician, that I am half-inclined to accept the theory that it is the man rather than medicine that heals. For my own part," he says, "when I am sick, I want my physician; but I am usually more benefited by his presence than by his prescriptions."

This secret, self-curing principle we all have in a greater or less degree, and only needs cultivation. *Authoritative assurance* is the remedy in most functional diseases, which relieves the morbid condition of the mind, and saves the body.

